# Crosstalk between arginine, glutamine, and the branched chain amino acid metabolism in the tumor microenvironment

**DOI:** 10.3389/fonc.2023.1186539

**Published:** 2023-05-19

**Authors:** Tanner J. Wetzel, Sheila C. Erfan, Lucas D. Figueroa, Leighton M. Wheeler, Elitsa A. Ananieva

**Affiliations:** Ananieva Laboratory, Biochemistry and Nutrition Department, Des Moines University, Des Moines, IA, United States

**Keywords:** glutamine, arginine, leucine, isoleucine, valine, TME, metabolism

## Abstract

Arginine, glutamine, and the branched chain amino acids (BCAAs) are a focus of increased interest in the field of oncology due to their importance in the metabolic reprogramming of cancer cells. In the tumor microenvironment (TME), these amino acids serve to support the elevated biosynthetic and energy demands of cancer cells, while simultaneously maintaining the growth, homeostasis, and effector function of tumor-infiltrating immune cells. To escape immune destruction, cancer cells utilize a variety of mechanisms to suppress the cytotoxic activity of effector T cells, facilitating T cell exhaustion. One such mechanism is the ability of cancer cells to overexpress metabolic enzymes specializing in the catabolism of arginine, glutamine, and the BCAAs in the TME. The action of such enzymes supplies cancer cells with metabolic intermediates that feed into the TCA cycle, supporting energy generation, or providing precursors for purine, pyrimidine, and polyamine biosynthesis. Armed with substantial metabolic flexibility, cancer cells redirect amino acids from the TME for their own advantage and growth, while leaving the local infiltrating effector T cells deprived of essential nutrients. This review addresses the metabolic pressure that cancer cells exert over immune cells in the TME by up-regulating amino acid metabolism, while discussing opportunities for targeting amino acid metabolism for therapeutic intervention. Special emphasis is given to the crosstalk between arginine, glutamine, and BCAA metabolism in affording cancer cells with metabolic dominance in the TME.

## 1 Introduction

Recent advances in our understanding of the interactions between cancer and immune cells strongly suggest the outcome of the anti-tumor T cell response is dictated by the nutrient availability and the flexibility of cancer and T cell metabolism (1–3). Cancer cells remodel their metabolism to escape immune surveillance in the TME creating nutrient-depleted TME with dysfunctional and exhausted T cells (4, 5). Amino acid deprivation is one of the signatures of nutrient-deprived TME.

Arginine, glutamine, and the BCAAs are needed to support the increased biosynthetic and bioenergetic demands of the growing tumor and the incoming tumor infiltrating lymphocytes (TILs) (6–8). These amino acids interconnect at several metabolic steps. Breakdown of BCAAs to branched chain keto acids (BCKAs) releases glutamate, which is the precursor for glutamine (9). Glutamine is converted into ornithine, which is the precursor of arginine (10). Arginine and ornithine are precursors for polyamine synthesis, which is upregulated in cancer and immune cells (11). The depletion of glutamine, arginine or the BCAAs in the TME, alone or in combination, may impact the ability of TILs to eliminate cancer cells. However, TILs and cancer cells share similar requirements for these amino acids, creating a practical conundrum regarding nutrient-based cancer treatments (12, 13). This review provides an overview of glutamine, arginine and the BCAAs based on recent discoveries in the context of TME and the challenges associated with future therapeutic approaches.

## 2 Overview of arginine

### 2.1 Arginine uptake and metabolism in mammalian cells

**Figure 1:**
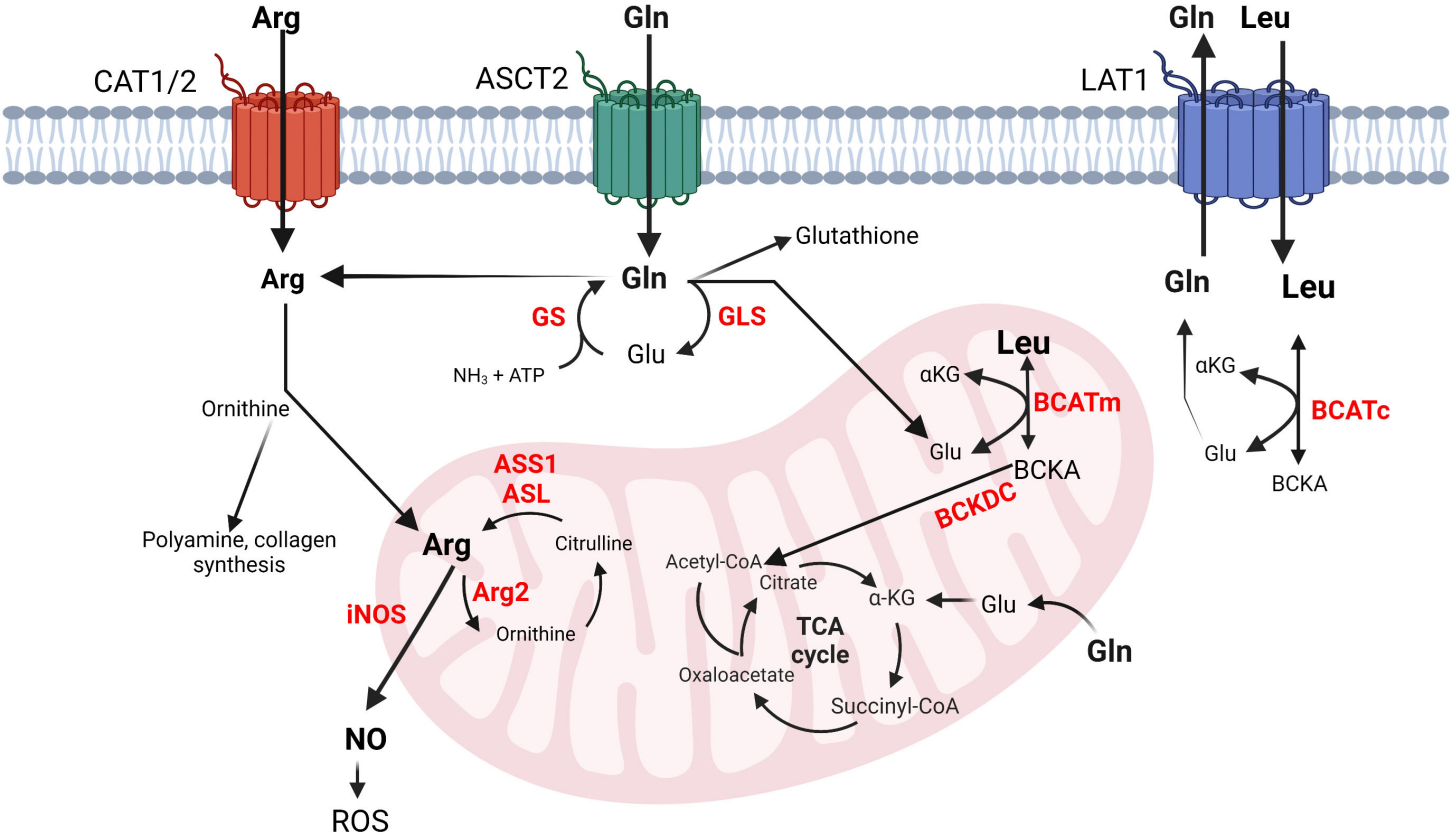
Simplified schematics of glutamine, arginine and BCAA metabolic interconnections in cancer and immune cells. Left to right: Arginine transportation is assisted by CAT. Arginine can be converted into ornithine, polyamines, collagen, or nitric oxide (NO). Glutamine enters the cells via ASCT2 and is converted into glutamate, glutathione, arginine, or nucleotides (not shown) or it may exit the cells via LAT1, which transfers leucine in exchange for glutamine. Leucine is converted into its corresponding BCKA in the cytosol or in the mitochondria. Arginine and glutamine (not shown) can be also synthesized in mitochondria. Glutamine and the BCAAs contribute to energy production by feeding into the TCA cycle. The metabolism of BCAAs is illustrated with leucine. The enzyme names are given in red. arginine, Arg, glutamine; Gln, glutamate; Glu, glutamine synthase; GS, glutaminase; GLS, arginosuccinate synthase 1; ASS1, arginosuccinate lyase; ASL, inducible nitric oxide synthase; iNOS, arginase 2; Arg2, leucine; Leu, α-ketoglutarate; αKG, cytosolic and mitochondrial branched chain aminotransferase BCATc and BCATm, branched chain keto acids; BCKAs, reactive oxygen species; ROS.

Dietary intake and protein degradation are the main sources of arginine for growing children. Postnatally, humans synthesize arginine via the intestinal-renal axis. This interorgan process includes the synthesis of citrulline by the small intestines and its absorption by the kidneys where citrulline is converted to arginine by argininosuccinate synthase 1 (ASS1) and lyase (ASL) (14). Once released in the circulation, arginine enters cells preferentially via cationic amino acid transporters (CATs) existing in eight different isoforms, each with different tissue distribution (**Figure 1**) (15). Inside the cells, arginine is incorporated into new protein, or used for polyamine and collagen synthesis, or as an activator of the mammalian target of rapamycin (mTOR) (**Figure 1**) (16). Thus, arginine availability is crucial for maintaining physiological cell function.

Arginine catabolism includes the urea cycle and nitric oxide (NO) production. The urea cycle comprises five enzymatic reactions that occur within the liver. Carbamoyl phosphate synthetase 1 (CPS1) incorporates ammonia into carbamoyl phosphate followed by formation of citrulline by ornithine transcarbamoylase (OTC), and argininosuccinate by ASS1. Arginine is then produced by ASL followed by hydrolysis by arginase 1 (Arg1) to urea and ornithine (16). Arg1 is a cytosolic enzyme expressed in the liver; however, humans express mitochondrial arginase, Arg2, in most tissues (17). During NO synthesis, nitric oxide synthases (NOS) catalyze the oxidation of arginine to NO and citrulline (**Figure 1**) (18). Mammals have three NOS isoforms, NOS1-3. NOS2 is the inducible and prevalent isoform in immune cells (iNOS) (19). The mononuclear myeloid-derived suppressor cells (M-MDSCs) rely on iNOS to drive immunosuppression (20). High expression of iNOS in M-MDSCs cells releases NO, which is converted into reactive oxygen species (ROS) causing DNA damage and promoting tumor growth (21).

### 2.2 Cancer and immune cells have high demands for arginine

Arginine is conditionally essential in patients with severe trauma, compromised immune system, or cancer cachexia. Under these disease states, the demand for arginine exceeds its endogenous production (22, 23).

Defective arginine synthesis (arginine auxotrophy) is a common occurrence in cancer cells. It primarily associates with a deficiency in ASS1 (24). To persist in the TME, CD4^+^ and CD8^+^ T cells must maintain adequate arginine concentrations. Arg2-deficient CD8^+^ T cells display enhanced cytotoxic activity against murine melanoma B16-OVA and colon adenocarcinoma MC38-OVA (25). The Arg2-deficent CD8^+^ T cells have improved effector function as seen by increased perforin, granzyme, IFN-γ and IL-2 (25). Alternatively, Arg2-specific human CD8^+^ T cells recognize Arg2-expressing regulatory T cells (Tregs), suggesting a naturally existing immunomodulatory potential of CD8^+^ T cells to remove immune suppression by targeting Tregs with high Arg2 expression (26). Similarly, Arg1-specific T cells target Arg1-expressing myeloid cells (27). In another study, bone marrow derived dendritic cells (BMDCs) and peritoneal macrophages synthesize arginine via ASL and ASS1 and supply CD4^+^ T cells with arginine (28). Studies with colorectal cancer patients failed to support the hypothesis that supplementation with arginine reduces the frequency of immunosuppressive M-MDSCs and polymorphonuclear myeloid-derived suppressor cells (PMN-MDSCs) but increases the frequency of CD4^+^ T cells. Thus, while arginine deficiency contributes to immunosuppression, systemic arginine supplementation alone does not restore immune system activity (29).

The rest of the urea cycle enzymes, CPS1 and OTC are studied to a lesser extent in cancer and immune cells (30). Cancer cells upregulate CPS1 to prevent ammonia buildup. A small-molecule inhibitor of CPS1 (H3B-120) that blocks CPS1 activity in human hepatocytes might be valuable for future therapeutic approaches (31). In contrast to CPS1, OTC is downregulated in cancer cells leading to accumulation of ammonia. Cancer cells can recycle ammonia for amino and nucleic acid synthesis (32). Lastly, a virus-induced metabolic reprogramming of mouse liver, results in transcriptional repression of the OTC and ASS1 genes leading to decreased arginine but increased ornithine concentrations in the circulation, which in turn suppresses virus-specific CD8^+^ T cells (33).

## 3 Overview of glutamine

### 3.1 Glutamine metabolism and transport in mammalian cells

Glutamine is the most abundant non-essential amino acid within human plasma. It contributes to nucleic acid (34) and protein synthesis (35), cellular response to ROS (36), and energy production through the TCA cycle (37). It is conditionally essential for proliferating cells during high demand, where endogenous synthesis is insufficient to support cellular homeostasis (38). Glutamine synthase (GS) generates glutamine from glutamate and ammonia (34). This reaction facilitates interorgan ammonium and glutamate transport, prevents toxic encephalopathy and blood acidification (35). Glutamine hydrolysis to glutamate and ammonia is facilitated, in part, by glutaminase-1 (GLS-1) in the kidney and glutaminase-2 (GLS-2) in the liver. Different transport systems specialize in assisting glutamine import and export by the cells. Among them are the sodium-dependent transporter ASCT2 (Solute Carrier 1a5, Slc1a5) and the sodium-independent antiporter Slc3a2 that work together with Slc7a5 (also known as L-type amino acid transporter 1, LAT1) to exchange glutamine for leucine (**Figure 1** **)**. These transporters have vast tissue distribution, but most notably they are overexpressed in immune and cancer cells (12, 36, 37).

### 3.2 Cancer and immune cells reliance on glutamine

Cancer reliance on glutamine is established in tumors throughout the body, including pancreatic (39), prostate (40), breast (41), and liver (42) cancers. Increased expression of ASCT2 and GLS are found in squamous cell carcinoma, adenocarcinoma, and neuroendocrine lung tumors (43). Such increases in the expression of ASCT2 and GLS are linked to tumors with aberrant oncogene c-MYC (40, 44). With a growing dependence on exogenous glutamine, tumor cells exhibit “glutamine addiction”. Glutamine addiction prevents cells from relying on endogenous glutamine synthesis and leads to cell death in glutamine free environments (45).

Similarly, immune cells rely on glutamine to sustain homeostasis and execute proper functions. A blockage of glutamine metabolism by DON (6-diazo-5-oxy-L-norleucine), or its modified prodrug JHU-083, causes a shift of CD8^+^ T cells towards a long-lived memory state and increases their tumor infiltration potential and survival in the TME (46–48). A loss of GLS halts Th17 differentiation but promotes the expression of Tbet and stimulates Th1 and CD8^+^ T cells. A long-term loss of GLS correlates with an impaired Th17 immune response, yet a transient loss of GLS promotes Th17, but restricts Th1 and CD8^+^ T cell effector differentiation (49). In a glutamine-depleted environment, activated CD8^+^ T cells produce significantly less IFN-γ and TNF-α (50). Selective GLS inhibition by CB-839, Telaglenastat, impairs the clonal expansion and activation of CD8^+^ T cells in the context of combinatorial anti-PD-1 treatment (51). In glutamine-addicted clear cell renal cell carcinoma (ccRCC), tumor-associated macrophages (TAMs) shift to M2 (immunosuppressive phenotype) promoting a pro-tumor environment. Such TAMs produce IL-23 in the context of hypoxia (HIF-α activation), activating Tregs (52). Taken together, glutamine metabolism plays an important role in T cell activation and function.

## 4 Overview of the branched chain amino acids

### 4.1 BCAA metabolism and transport in mammalian cells

The BCAAs (leucine, isoleucine, and valine) are supplemented through the diet to mammalian cells. BCAAs make up ~35% of the essential amino acids in the blood (53). The BCAAs are important nutrients under physiological and pathological conditions (54). They are nitrogen donors to glutamate and alanine and stimulate protein synthesis in the muscle (55). In the brain, the BCAAs maintain the glutamate-glutamine interconversions by engaging in “glutamate-BCAA” cycles between neurons and astrocytes (56). BCAAs trigger insulin release from the pancreatic β-islets; however, chronically elevated plasma BCAAs are a common clinical finding in patients with Type 2 Diabetes and Cardiovascular Disease (56, 57).

BCAAs travel across the plasma membranes utilizing the heterodimeric transporter Slc7a5/Slc3a2. As stated earlier, this transporter works in antiport with Slc1a5 where glutamine efflux proceeds BCAA influx (6, 58). Once inside the cells, BCAAs are incorporated into protein or subjected to degradation by the cytosolic branched chain aminotransferase, BCATc (6). Alternately, the BCAAs enter the mitochondria, assisted by the Scl24a44 transporter, to become subjected to degradation by the mitochondrial BCATm (59). BCATc and BCATm catalyze the reversible transamination of the BCAAs to their corresponding BCKAs, which are subjected to irreversible oxidative decarboxylation by the mitochondrial branched chain alpha-ketoacid dehydrogenase complex (BCKDC). Following this step, each BCAA commits to their unique degradation pathways releasing propionyl-CoA, acetoacetate, or acetyl-CoA that feed into the TCA cycle or other pathways (**Figure 1**) (60).

### 4.2 BCAAs support cancer growth but they are also essential for proper immune function

BCAAs are important for sustainable tumor growth. The growing tumor obtains BCAAs from the circulation or the tissues surrounding it. Positive association between elevated plasma BCAAs and the risk of colorectal adenoma and pancreatic adenocarcinoma are reported in human patients but controversial in animal studies (61–65). High plasma concentrations of BCAAs, due to disruption in BCAA metabolism, or dietary supplementation with BCAAs, are associated with delayed onset of lymphoma, or suppression of breast cancer in mice (63, 64). In contrast, mice subjected to a diet high in BCAAs, have increased incidences of pancreatic ductal adenocarcinoma (PDAC) (66). Elevated BCAA metabolism at the BCAT step is implicated in the onset of many cancers including glioblastoma (53) myeloid leukemia (54) lymphoma (50) lung (55), gastric (56), pancreatic (57) and breast cancers (58).

To a lesser extent, BCAAs and their metabolism are studied in immune cells. Leucine is indispensable for T cell activation as insufficient leucine prevents clonal expansion to Th1, Th17 and CD8^+^ T cells (37). Mice deficient of Slc3a2 in Foxp3^+^ Tregs, generate a low number of Foxp3^+^ Tregs and fail to suppress intestinal inflammation (67). CD4^+^ T cells, deficient in BCATc or BCATm, have higher glycolytic capacity, improved oxygen consumption and increased capacity to secrete IFNγ (6, 68). Studies with BCATc in human macrophages identified a non-catalytic role for BCATc in the metabolic events associated with fragmented TCA cycle (69, 70). It remains to be further established whether the non-enzymatic function of BCATc represents a universal mechanism to regulate cellular metabolism.

## 5 Discussion

### 5.1 The interconnected network between arginine, glutamine and the BCAAs in TME

Rapidly dividing cancer cells are forced to reprogram their metabolism to ensure long term survival and metastatic growth. Their major opponents, the effector Th1 and CD8^+^ T cells, must also reprogram metabolism to embrace the harsh TME. However, these functionally unrelated cells have similar demands for nutrients, including amino acids (71, 72).

Numerous reports have demonstrated uptake of glutamine, arginine, and the BCAAs is upregulated in cancer and activated Th1 and CD8^+^ T cells (6, 73–75). There is a high redundancy in transport preference for these amino acids, making current approaches to target amino acid uptake particularly challenging (6, 76). Ovarian cancer cells, CD4^+^ and CD8^+^ memory T cells, and M0 macrophages overexpress the arginine CAT1 transporter. Silencing CAT1 in the ovarian cancer cells significantly reduces the concentration of arginine but lowers the concentrations of BCAAs (77). The uptake of glutamine by human breast HCC1806 cancer cells, deficient in ASCT2, is sensitive to the inhibition of leucine uptake when LAT1 is targeted by JPH203 (78). This suggests that LAT1 plays a role as a rescue transporter for glutamine. In breast cancer biopsies, high LAT1 expression is associated with invasive breast cancer where LAT1 overexpression positively correlates with the expression of the estrogen receptor (ER) and the programmed death ligand-1 (PD-L1) (79). LAT1 is highly expressed in malignant skin lesions (80) and in cells from patients with skin disorders (81). Increased LAT1 expression is observed in keratinocytes and dermal infiltrating lymphocytes of patients with psoriasis, where LAT1 expression is upregulated by IL-23 and IL-1b (81). Thus, scientific evidence exists to support the notion of high reliance of malignant and non-malignant cells on amino acid transporters specializing in the uptake of arginine, glutamine, and the BCAAs. Because these transporters exert overlapping functions, their targeting may impact the uptake of more than one amino acid in clinical trials.

**Figure 2:**
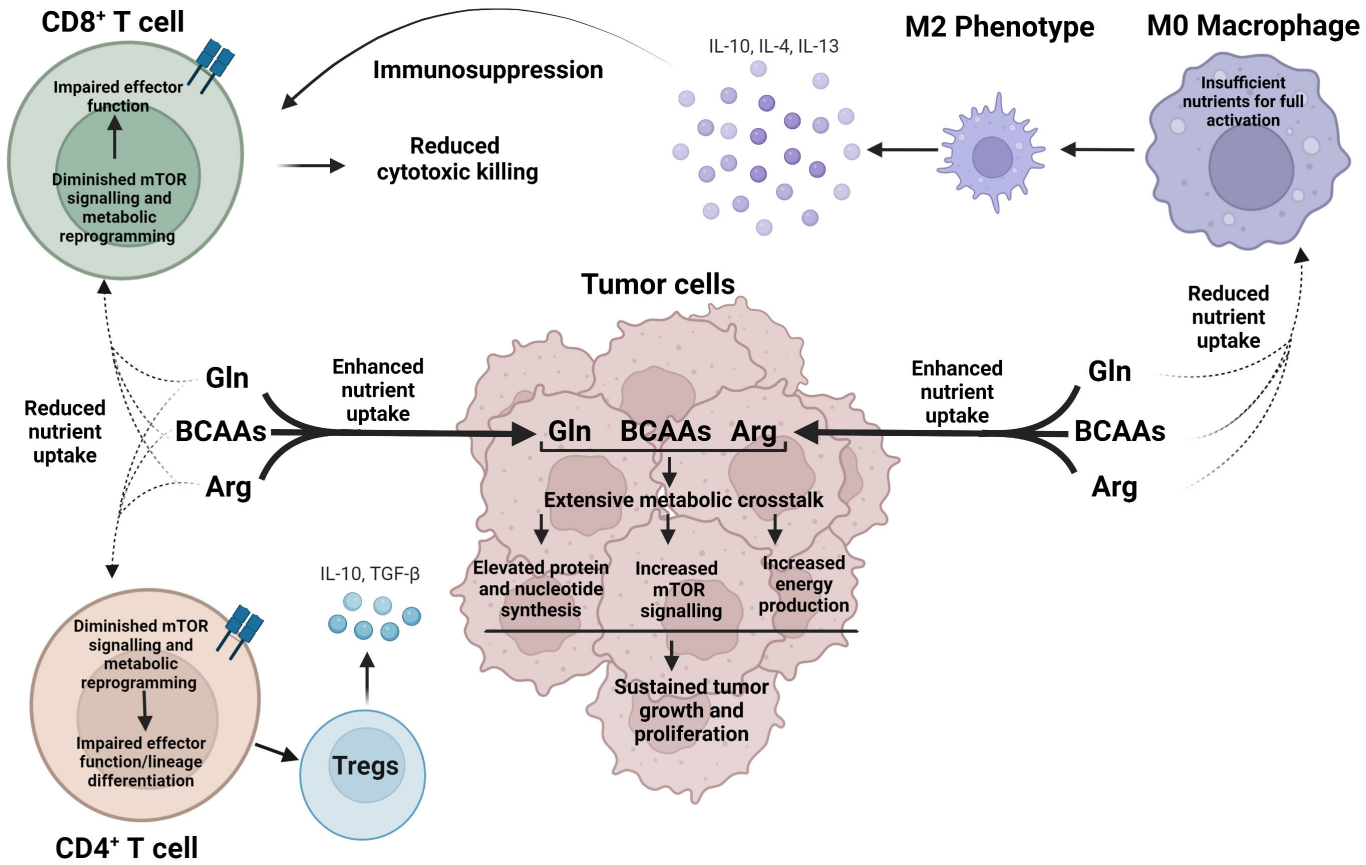
Cancer cells exert metabolic dominance over immune cells within the TME to avoid detection and destruction. In a nutrient-depleted TME, cancer cells preferentially uptake arginine, glutamine, and BCAAs, which undergo vast, interconnected metabolic pathways to produce essential biosynthetic precursors to support rapid cancer growth, as well as activate mTOR signaling. Oppositely, reduced nutrient uptake of arginine, glutamine, and the BCAAs voids CD4^+^ and CD8^+^ T cells of essential nutrients and diminishes mTOR signaling leading to impaired effector function and aberrant lineage commitment. As a result, immune cells, such as M2 macrophages and Tregs cells, are generated, which in turn release immunosuppressive cytokines, promoting an environment for cancer growth. Arg, arginine; Gln, glutamine; BCAAs Branched chain amino acids.

Most of arginine, glutamine and the BCAAs are delivered to the TME for incorporation in new protein. However, 20-25% are degraded or used to stimulate signal transduction cascades, such as mTOR pathway (72). Such distribution is necessary to supply the cells with fuel and precursor metabolites for purine, pyrimidine, or polyamine biosynthesis (**Figure 2**) (82–85). The intracellular concentrations of these amino acids, however, fluctuate based on shared metabolic precursors and enzymatic reactions. A global deletion of BCATm leads to a reduction in lymphoma burden, which correlates with elevated concentrations of BCAAs, but reduced concentrations of glutamine (64). In a non-small cell lung carcinoma (NSCLC), nitrogen derived from BCAA transamination supports glutamine and nucleotide synthesis via the glutamine-purine-pyrimidine axis. However, such reliance on nitrogen from BCAAs is not observed in PDAC (86). In contrast, BCATc selective inhibition, but not changes in the BCAAs, results in upregulation of genes involved in the transport of glutamate and the conversion of glutamate into glutathione in human macrophages (70). Similarly, mouse embryonic fibroblasts, grown in a glutamine-depleted environment, show a significant increase in arginine, but not in BCAAs. The arginine levels balance off, while the levels of BCAAs increase when *TP53* is deleted. The authors thus identified the tumor suppressor p53 as an important transcriptional regulator of arginine uptake during a pro-survival response to glutamine-induced metabolic stress (87). In the TME, restricting glutamine or glutamine-dependent purine and pyrimidine synthesis shifts CD4^+^ T cells toward Tregs but this shift is abolished if GS is inhibited. GS is described as de-repressed under low glutamine, or nucleotide starvation (88). Arginine is a precursor of polyamines and targeting enzymes such as Arg1, can impact the synthesis of polyamines in the TME. Polyamines exert immunosuppressive effects, promoting tumor growth (83). Arg1 is overexpressed in dendritic cells and represents one of the immune checkpoints in the TME (11). Dendritic cells may deprive the TME of arginine causing T cell exhaustion (83).

Lastly, arginine, glutamine and the BCAAs activate complex 1 of mTOR in cancer and immune cells. Nutrient sensing via mTOR is essential for growth and survival; however, in the context of TME, this is yet another mechanism cancer and immune cells exploit to compete for nutrients (**Figure 2**). mTOR signaling is dysregulated in cancer cells, while T cell function requires upregulation of mTOR (89–91). While leucine is the most potent activator of mTOR as reviewed in (6), glutamine and arginine are other stimulators of mTOR signaling. mTOR sensing may occur via Rag-GTPase-dependent and independent pathways and may engage different protein targets (92, 93). Leucine-driven activation of mTOR includes GATOR1-2, Sestrin2, and SAR1B and follows the Rag-GTPase dependent mechanism (94, 95). Arginine cannot bind Sestrin 2 or SAR1B but requires a lysosomal membrane protein SLC38A9 (96). Glutamine synergizes asparagine to activate mTOR signaling via Rag-GTPase independent mechanism (93). In summary, cancer and immune cells co-exist in the TME in a bidirectional metabolic relationship, influenced by the fluctuations in arginine, glutamine and the BCAAs.

### 5.2 Targeting arginine, glutamine and the BCAAs for cancer therapy

Because arginine, glutamine and the BCAAs are required for growth of cancer and immune cells, targeted deprivation or supplementation of these amino acids may lead to undesirable therapeutic effects (97). Selectively limiting the availability of these amino acids in tumor cells while supplying them to immune cells may help overcome this obstacle. Indeed, pharmacological inhibition of glutamine uptake by the ASCT2 inhibitor, V-9302, blocks glutamine uptake in triple negative breast cancer cells but not in CD8^+^ T cells. The CD8^+^ T cells adapt by upregulating a Na^+^/Cl^-^dependent neutral and cationic amino acid transporter ATB^0,+^ (98). A similar approach is used in keratinocytes from patients with psoriasis, where deleting LAT1 controls skin inflammation, while CD4^+^ T cells use alternative amino acid transporters (LAT2 and LAT3) (81). Lastly, pro-drugs, such as DRP104, target GLS-1 in tumors and cause CD8^+^ T cell-dependent tumor regression (94) Such approaches could potentially unleash the immune cells in destroying cancer cells in the TME.

The endurance of the chimeric antigen receptor T (CAR-T) cells in hematological and solid malignancies can be affected by amino-acid depleted TME. Induced expression of ASS1 in re-engineered CAR-T cells increases their proliferation without compromising their function (99).

A combinatorial therapy including multivesicular liposome technology, designed to supply arginine to melanoma tumors, and selective suppression of the CAT2 transporter, leads to arginine starvation of tumor cells but promotes the infiltration of CD8^+^ T cells in the TME (100). Similarly, a local therapy using nanoparticles to deliver poly(L-arginine) and hyaluronic acid to tumor-associated macrophages successfully induces tumor-suppressive M1 phenotype and leads to an increased iNOS expression in these cells (101).

Although still in their infancy, nanomaterials or liposome-based technologies could be expanded to deliver glutamine and BCAAs to CD8^+^ and CD4^+^T cells in the TME. In addition, new generations of CAR-T cells could be designed to competitively intake arginine, glutamine and BCAAs from the TME. Under such a scenario, systemic side effects should be minimal and can address the low therapeutic efficacy of the conventional cancer therapies.

## Author contributions

TW and EA designed the mini review. TW prepared the glutamine overview and **Figures 1**, **2**. SE prepared the arginine overview. LW and LF prepared the BCAA overview. EA compiled and edit the different sections. All authors contributed to the article and approved the submitted version.

## Glossary

**Table:**

BCAA	Branched chain amino acids
TME	Tumor microenvironment
TILs	Tumor infiltrating lymphocytes
BCKA	Branched chain keto acid
ASS1	Arginosuccinate synthase 1
ASL	Arginosuccinate lyase
CAT	Cationic amino acid transporter
NO	Nitric oxide
mTOR	Mammalian target of rapamycin
Arg1	Arginase 1
Arg2	Arginase 2
NOS	Nitric oxide synthase
M-MDSCs	Mononuclear myeloid-derived suppressor cells
BMDCs	Bone marrow derived dendritic cells
PMN-MDSCs	Polymorphonuclear myeloid derived suppressor cells
GS	Glutamine synthase
GLS-1	Glutaminase-1
GLS-2	Glutaminase-2
ASCT2	Alanine/Serine/Cysteine transporter
SLC	Sodium dependent transporter
LAT1	L-type amino acid transporter 1
L-DON	6-diazo-5-oxo-L-norleucine
NK cells	Natural killer cells
TAMs	Tumor associated macrophages
Treg	Regulatory T cells
HIF1α	Hypoxia inducible factor 1 α
BCATc	Cytosolic branched chain aminotransferase
BCATm	Mitochondrial branched chain aminotransferase
BCKDC	Mitochondrial branched chain alpha-keto acid dehydrogenase complex
PDAC	Pancreatic ductal adenocarcinoma
ER	Estrogen receptor
PD-L1	Programed death receptor ligand-1
NSCLC	Non-small cell lung carcinoma
CAR-T	Chimeric antigen receptor T cells
ccRCC	Clear cell renal cell carcinoma
